# Staurosporine synergistically potentiates the deoxycholate‐mediated induction of COX‐2 expression

**DOI:** 10.14814/phy2.12143

**Published:** 2014-08-28

**Authors:** Tohru Saeki, Haruka Inui, Saya Fujioka, Suguru Fukuda, Ayumi Nomura, Yasushi Nakamura, Eun Young Park, Kenji Sato, Ryuhei Kanamoto

**Affiliations:** 1 Laboratory of Molecular Nutrition, Kyoto Prefectural University, Kyoto, Japan; 2 Laboratory of Food Science, Kyoto Prefectural University, Kyoto, Japan; Laboratory of Physiological Function of Food, Division of Food Science and Biotechnology, Graduate School of Agriculture, Kyoto University, Gokasho, Uji, 611‐0011, Kyoto, Japan; Central Research Institute, Japan Clinic Co., Ltd, 10‐1 Kainichi‐choUzumasa, Ukyo‐ku, 616‐8555, Kyoto, Japan

**Keywords:** Bile acids, colorectal cancer cells, cyclooxygenase‐2, staurosporine

## Abstract

Colorectal cancer is a major cause of cancer‐related death in western countries, and thus there is an urgent need to elucidate the mechanism of colorectal tumorigenesis. A diet that is rich in fat increases the risk of colorectal tumorigenesis. Bile acids, which are secreted in response to the ingestion of fat, have been shown to increase the risk of colorectal tumors. The expression of cyclooxygenase (COX)‐2, an inducible isozyme of cyclooxygenase, is induced by bile acids and correlates with the incidence and progression of cancers. In this study, we investigated the signal transduction pathways involved in the bile‐acid‐mediated induction of COX‐2 expression. We found that staurosporine (sts), a potent protein kinase C (PKC) inhibitor, synergistically potentiated the deoxycholate‐mediated induction of COX‐2 expression. Sts did not increase the stabilization of *COX‐2 *mRNA. The sts‐ and deoxycholate‐mediated synergistic induction of COX‐2 expression was suppressed by a membrane‐permeable Ca^2+^ chelator, a phosphoinositide 3‐kinase inhibitor, a nuclear factor‐*κ*B pathway inhibitor, and inhibitors of canonical and stress‐inducible mitogen‐activated protein kinase pathways. Inhibition was also observed using PKC inhibitors, suggesting the involvement of certain PKC isozymes (*η*,* θ*,* ι*,* ζ*, or *μ*). Our results indicate that sts exerts its potentiating effects via the phosphorylation of p38. However, the effects of anisomycin did not mimic those of sts, indicating that although p38 activation is required, it does not enhance deoxycholate‐induced COX‐2 expression. We conclude that staurosporine synergistically enhances deoxycholate‐induced COX‐2 expression in RCM‐1 colon cancer cells.

## Abstract (precis)

e12143

The expression of COX‐2, an inducible isozyme of cyclooxygenase, correlates with the incidence and progression of cancers, and bile acids have been shown to induce COX‐2 expression. We investigated the signal transduction pathways involved in the bile‐acid‐mediated induction of COX‐2 expression, and we found that staurosporine, a potent PKC inhibitor, synergistically potentiated the deoxycholate‐mediated induction of COX‐2 expression. Staurosporine exerted its potentiating effects via the phosphorylation of p38, and the involvement of certain PKC isozymes was suggested.

## Introduction

Colorectal cancer is a major cause of cancer‐related death in western countries (Future Trends in Colorectal Cancer Research 1999; Key et al. 2002). Furthermore, the incidence of colorectal cancer in Japan has increased gradually since the 1950s, making it the second most common type of cancer in Japan (Matsuda et al. 2013). Diet is considered to be a major environmental factor that might be involved in the increased incidence of colorectal cancer (Sandler et al. 1993), and consumption of a western diet, which is high in fat, increases the risk of colorectal tumorigenesis. Bile acids are secreted in response to fat ingestion and contribute to the risk of colorectal tumors (Weisburger et al. 1983; Bayerdorffer et al. 1993; Debruyne et al. 2001; Jenkins et al. 2004; Bernstein et al. 2009).

Bile acids are surfactants synthesized from cholesterol in the liver that aid in the digestion of fats and fat‐soluble nutrients. The primary bile acids, cholic acid, and chenodeoxycholic acid (CDC), are conjugated with taurine or glycine and then secreted into the duodenum. Bile acids are reabsorbed at the distal end of the ileum via a sodium‐dependent transporter (SLC10A2) and returned to the liver (Saeki et al. 2002, 2013). Two to five percent of bile acids remain in the colon, where they are converted to secondary bile acids through deconjugation and 7*α*‐dehydroxylation, which increases their hydrophobicity. Secondary bile acids include deoxycholic acid (DC) and lithocholic acid, which are produced from cholic acid and CDC, respectively.

Secondary bile acids and the hydrophobic primary bile acid CDC have been associated with an increased risk of cancer (Reddy et al. 1977; Bayerdorffer et al. 1993; Mahmoud et al. 1999; Jenkins et al. 2004; Bernstein et al. 2009). In a previous study, we found that the prevention of bile acid reabsorption by surgical removal of the ileum increased the incidence and size of colon tumors in rats that were fed DC (Kanamoto et al. 1999). An increased concentration of hydrophobic bile acids has also been reported in the feces of patients with intestinal adenomatous polyps (Reddy et al. 1976; Imray et al. 1992). Although the precise mechanism by which bile acids contribute to the formation of tumors is unclear, the induction of cyclooxygenase (COX)‐2 expression by bile acids is thought to be involved.

Cyclooxygenase, also known as prostaglandin G/H synthase, activates the synthesis cascade for the production of prostanoids from eicosapolyenoic acids, such as arachidonic acid. The COX‐1 protein is constitutively expressed in most tissues, and COX‐3, a splice variant of COX‐1, is abundant in the cerebral cortex and heart (Chandrasekharan et al. 2002). Although the COX‐2 protein is not expressed in most healthy tissues, COX‐2 expression is enhanced at both the transcriptional and posttranscriptional levels by growth factors, tumor promoters, oncogenes, inflammation mediators, and carcinogens (Kujubu et al. 1991; Jones et al. 1993; DuBois et al. 1994; Inoue et al. 1995; Subbaramaiah et al. 1996; Fernau et al. 2010; McElroy et al. 2012; Dusaban et al. 2013; MacKenzie et al. 2013).

In ovarian cancer cells, insulin‐like growth factor‐1 induces the expression of COX‐2 at the transcriptional level and stabilizes the *COX‐2* mRNA via the phosphoinositide 3‐kinase (PI3K), mitogen‐activated protein kinase (MAPK), and protein kinase C (PKC) pathways (Cao et al. 2007). In human keratinocytes and synovial fibroblasts, ultraviolet B (UVB) radiation and interleukin (IL)‐1*β* stimulate COX‐2 expression by stabilizing the *COX‐2* mRNA via Ras, p38, and C/EBP*β* (Faour et al. 2001; Fernau et al. 2010). The expression of COX‐2 correlates with the incidence and progression of adenomas and adenocarcinomas, and the inhibition of COX‐2 activity has been shown to reduce the risk of cancer and improve the efficacy of cancer treatments (Eberhart et al. 1994; Ding et al. 2001; Gupta and Dubois 2001; Shaheen et al. 2002; Dannenberg and Subbaramaiah 2003; Ricchi et al. 2003; Subbaramaiah and Dannenberg 2003; Chun and Surh 2004).

The induction of COX‐2 expression by bile acids has been demonstrated in various types of cells. In duodenal reflux, bile acids enhance COX‐2 expression in the esophageal mucosa through activator protein (AP)‐1 and nuclear factor‐*κ*B (NF‐*κ*B), and increased COX‐2 expression has been associated with esophageal adenocarcinoma (Zhang et al. 1998, 2000; Shirvani et al. 2000; Looby et al. 2009; Burnat et al. 2010). In colorectal cancer cells, the peroxisome proliferator‐activated receptor‐*α* (PPAR*α*) and the cyclic AMP‐responsive element have been shown to be involved in the bile‐acid‐mediated transcriptional activation of the *COX‐2* gene (Oshio et al. 2008). Bile acids have also been shown to enhance COX‐2 expression in intestinal epithelial cells by stabilizing *COX‐2* mRNA (Zhang et al. 2000).

In this study, we investigated the signal transduction pathways involved in DC‐induced COX‐2 expression using staurosporine (sts), an alkaloid isolated from *Streptomyces staurosporesa*. Sts functions as a membrane‐permeable PKC inhibitor that competes with ATP at the catalytic site of PKC with a subnanomolar IC_50_ (Gschwendt et al. 1996), and also inhibits certain other kinases at higher concentrations (Meggio et al. 1995). We found that pretreatment with sts synergistically enhanced DC‐induced COX‐2 expression. Our results suggest that, under certain conditions, bile acids may induce the expression of COX‐2 at far higher levels than those previously reported. We also investigated the signaling pathways involved in the synergistic effect of sts on DC‐induced COX‐2 expression, and found that sts acts via the p38 pathway.

## Materials and Methods

### Reagents and antibodies

Sts (Sigma‐Aldrich, St. Louis, MO), bisindolylmaleimide IX (Ro; Ro 31‐8220, Calbiochem Merck, Darmstadt, Germany), bisindolylmaleimide I (GF; GF 109203X or Gö 6850, Sigma‐Aldrich), the PI3K inhibitor LY294002 (LY; Calbiochem Merck and Cayman Chemical, Ann Arbor, MI), the p38 MAPK inhibitor SB202190 (SB; Sigma‐Aldrich), the MAPK kinase 1/2 (MEK) inhibitor U0126 (Sigma‐Aldrich, Calbiochem, and Cell Signaling Technology, Danvers, MA), the Jun‐amino‐terminal kinase 1/2/3 inhibitor SP600125 (SP; Sigma‐Aldrich and Santa Cruz Biotechnology, Santa Cruz, CA), *O*,*O*′‐bis(2‐aminophenyl)ethyleneglycol‐*N*,*N*,*N*′,*N*′‐tetraacetic acid, tetraacetoxymethyl ester (BAPTA‐AM; Nacalai Tesque, Kyoto, Japan), anisomycin (Calbiochem Merck), BAY 11‐7082 (BAY; Calbiochem Merck), and actinomycin D (ActD; Sigma‐Aldrich) were dissolved in dimethyl sulfoxide (DMSO). The concentration of the vehicle (DMSO) in culture media was maintained at <0.5%, and was uniformly adjusted among the culture dishes in each set of experiments. A 100‐mmol/L sodium deoxycholate (Sigma‐Aldrich) solution was prepared in sterile water. The protease inhibitor cocktail was purchased from Nacalai Tesque (Kyoto, Japan). The rabbit anti‐COX‐2 polyclonal antibody and the mouse anti‐COX‐2 monoclonal antibody were purchased from Cell Signaling Technology and Cayman Chemical, respectively. The rabbit anti‐p38 (total) and anti‐phospho‐p38 (Thr180/Tyr182) polyclonal antibodies were purchased from R&D Systems (Minneapolis, MN). The rabbit anti‐p44/42 MAPK (total extracellular signal‐regulated protein kinase [ERK] 1/2) and anti‐phospho‐ERK1/2 (Thr202/Tyr204) polyclonal antibodies were purchased from Cell Signaling Technology. The rabbit anti‐*β*‐actin polyclonal antibody was purchased from BioVision (Mountain View, CA) or Imgenex (San Diego, CA). The rabbit anti‐glyceraldehyde 3‐phosphate dehydrogenase (GAPDH) monoclonal antibody was purchased from Cell Signaling Technology. The peroxidase‐labeled anti‐rabbit IgG and anti‐mouse IgG secondary antibodies were purchased from Vector Laboratories (Burlingame, CA) or Nacalai Tesque.

### Cell culture methods

The RCM‐1 colon cancer cell line, originally derived from a well‐differentiated rectum adenocarcinoma of a 73‐year‐old woman (Kataoka et al. 1989), was obtained from Dr. H. Kataoka of the University of Miyazaki (Miyazaki, Japan). The RCM‐1 cells were grown at 37°C in a humidified atmosphere with 5% CO_2_ in a 1:1 mixture of RPMI‐1640 medium and Ham F12 nutrient mixture supplemented with 10% fetal bovine serum (FBS), 1 mmol/L l‐glutamine, and 10 *μ*g/mL gentamicin. The HT‐29 colon cancer cells were grown at 37°C in a humidified atmosphere with 5% CO_2_ in McCoy 5A medium supplemented with 10% FBS and 10 *μ*g/mL gentamicin.

### Induction of COX‐2 expression and western blotting

Four days before induction, approximately 1.5 × 10^6^ RCM‐1 cells or 0.6 × 10^6^ HT‐29 cells were seeded in a 3.5‐cm dish and incubated for 3 days. On the day before the induction, the medium was replaced with serum‐free medium, and the cells were incubated for 24 h. The cells were pretreated with 10, 20, 50, 63, or 100 nmol/L sts or vehicle control (0.05% DMSO) in the serum‐free medium for 30 min. For the DC treatment, the cells were incubated in 100 *μ*mol/L DC in serum‐free medium with or without sts for 24 h. The cells were washed with phosphate‐buffered saline and lysed by sonication in a buffer containing 10 mmol/L Tris HCl (pH 7.4), 0.1 mmol/L ethylenedinitrilotetraacetic acid (EDTA), 0.1% sodium dodecyl sulfate (SDS), 1 nmol/L 4‐(2‐aminoethyl) benzenesulfonyl fluoride hydrochloride, 800 nmol/L aprotinin, 50 *μ*mol/L bestatin, 15 *μ*mol/L l‐*trans*‐epoxysuccinyl‐leucylamido(4‐guanido)butane (E‐64), 20 *μ*mol/L leupeptin hemisulfate, and 10 *μ*mol/L pepstatin A. Aliquots of the whole cell lysates containing 20–40 *μ*g of protein were subjected to SDS‐polyacrylamide gel electrophoresis using a 10% polyacrylamide gel. The resolved protein bands were electrophoretically transferred onto a polyvinylidene difluoride membrane, and western blotting was performed using an anti‐COX‐2 antibody and the various other primary antibodies. Primary antibody reactivity was detected using the appropriate secondary antibody.

### Inhibition of mRNA synthesis

To measure the intracellular degradation of mRNA, the cells were treated with ActD, an inhibitor of DNA‐primed RNA synthesis. The RCM‐1 cells were pretreated with 10 nmol/L sts or the vehicle control for 30 min, followed by treatment with 100 *μ*mol/L DC for 4 h. The medium was replaced with a fresh medium containing 2 *μ*g/mL ActD, and the levels of *COX‐2* mRNA were determined by quantitative reverse transcription and polymerase chain reaction (PCR) at the various time points.

### Quantitative analysis of COX‐2 mRNA expression

Total RNA was isolated from RCM‐1 cells using RNAiso (Takara Bio, Shiga, Japan), according to the manufacturer's instructions. First‐strand cDNA was synthesized by reverse transcriptase using oligo dT primers, random hexamer primers, and the PrimeScript Enzyme Mix I (Takara Bio). The cDNAs were used as templates for real‐time PCR using SYBR Premix Ex Taq II (Takara Bio), according to the manufacturer's instructions. Real‐time PCR amplification of the *β*‐actin mRNA was used as an endogenous control. The *COX‐2* gene‐specific primers used were 5′‐ATTGAGTACCGCAAACGCTTTA‐3′ (forward) and 5′‐TTCCAACTCTGCAGACATTTCC‐3′ (reverse), and the *β*‐actin gene‐specific primers were 5′‐CTACAATGAGCTGCGTGTGG‐3′ (forward) and 5′‐ATGGCTACGTACATGGCTGG‐3′ (reverse). The final concentration of each primer was 400 nmol/L. Real‐time PCR was performed using a Rotor‐Gene Q instrument (Qiagen, Hilden, Germany), according to the manufacturer's instructions. Thermal cycling was performed as follows for 50 (*COX‐2*) or 35 (*β*‐actin) cycles: denaturation at 95°C for 4 sec; annealing at 56°C (*COX‐2*) or 62°C (*β*‐actin) for 20 sec; extension at 72°C for 30 sec. The level of *COX‐2* mRNA detected was normalized to that of *β*‐actin in the individual samples.

### Densitometry and statistical analysis

The relative intensities of protein bands in the western blots were analyzed using ImageJ software (http://rsb.info.nig.gov/ij/). The effects of the various treatments were compared using a one‐way analysis of variance (ANOVA), followed by the Tukey‐Kramer HSD test or the Dunnett test using JMP software (SAS Institute, Cary, NC).

## Results

**Figure 1.**
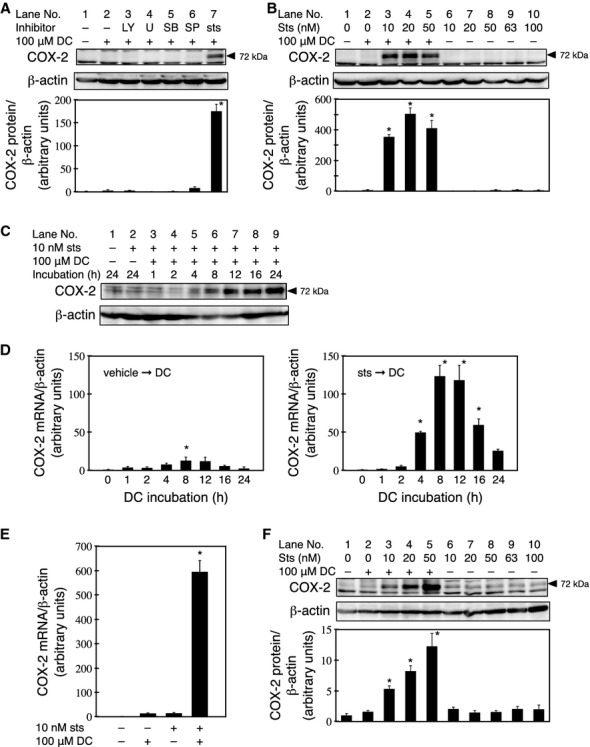
Staurosporine mediates synergistic potentiation of DC‐induced COX‐2 expression. (A) RCM‐1 cells were pretreated with 20 *μ*mol/L LY294002 (LY), 10 *μ*mol/L U0126 (U), 20 *μ*mol/L SB202190 (SB), 50 *μ*mol/L SP600125 (SP), 10 nmol/L staurosporine (sts), or vehicle control (0.13% DMSO) for 30 min, followed by a coincubation with each inhibitor and 100 *μ*mol/L DC for 24 h. COX‐2 protein levels were determined by western blotting. The membrane was stripped, and then reprobed using an anti‐*β*‐actin antibody. Densitometry was used to determine the relative intensities of the COX‐2 bands in the immunoblots. Relative intensities are expressed as the mean ± SE of three independent experiments (*P < 0.05 compared to the cells treated with vehicle control only). (B) RCM‐1 cells were pretreated with the indicated concentration of sts for 30 min, followed by a coincubation with each concentration of sts and 100 *μ*mol/L DC for 24 h. The levels of COX‐2 protein were determined by western blotting. The membrane was stripped, and then reprobed using an anti‐*β*‐actin antibody. Relative intensities are expressed as the mean ± SE of three independent experiments (*P < 0.05 compared to the cells treated with vehicle control only). (C) The RCM‐1 cells were pretreated with 10 nmol/L sts for 30 min. DC was added to give a final concentration of 100 *μ*mol/L, and incubation was continued for the indicated periods. The level of COX‐2 protein was determined by western blotting. (D) The RCM‐1 cells were pretreated with 10 nmol/L sts or vehicle control (0.05% DMSO) for 30 min. DC was added to give a final concentration of 100 *μ*mol/L, and incubation was continued for the indicated periods. The level of *COX‐2 *mRNA was determined by real‐time PCR. Each column and bar represents the mean ± SE determined of three independent experiments (*P < 0.05 compared to the mRNA level at 0 h). (E) The RCM‐1 cells were pretreated with 10 nmol/L sts or vehicle control (0.05% DMSO) prior to treatment with or without 100 *μ*mol/L DC for 4 h. The levels of *COX‐2 *mRNA were determined by real‐time PCR. Each column and bar represents the mean ± SE determined of three independent experiments (*P < 0.05, compared to the cells treated with vehicle control only). (F) HT‐29 cells were pretreated with the indicated concentration of sts for 30 min, followed by a coincubation with each concentration of sts and 100 *μ*mol/L DC for 24 h. The levels of COX‐2 protein were determined by western blotting. The membrane was stripped, and then reprobed using an anti‐*β*‐actin antibody. The relative intensities represent the mean ± SE of three independent experiments (*P < 0.05 compared to the cells treated with vehicle control only).

To identify the signal transduction pathways involved in DC‐induced COX‐2 expression, RCM‐1 cells were pretreated with various kinase inhibitors followed by treatment with 100 *μ*mol/L DC for 24 h. Only a low level of COX‐2 expression was detected after the DC treatment (Fig. 1A, lane 2). While the kinase inhibitors LY, U0126, SB, and SP did not exert a significant effect on COX‐2 expression (Fig. 1A, lanes 3–6), a 30‐min pretreatment with 10 nmol/L sts significantly increased the expression of COX‐2 (Fig. 1A, lane 7). Pretreatment with 50 or 100 nmol/L sts resulted in similarly high levels of COX‐2 expression, indicating that 10 nmol/L sts is sufficient to potentiate DC‐induced COX‐2 expression (Fig. 1B, lanes 3–5). Previous studies have shown that 63 nmol/L sts induces COX‐2 expression in rat peritoneal macrophages (Yamaki et al. 2000). However, we observed that treatment with 63 or 100 nmol/L sts in the absent of DC resulted in no significant increase in COX‐2 expression in RCM‐1 cells (Fig. 1B lanes 6–10). These results indicate that sts synergistically enhances DC‐mediated COX‐2 expression.

We next performed a time course experiment for sts‐DC‐mediated COX‐2 expression, and observed that the COX‐2 protein was detectable 4 h after the initiation of DC treatment, and that COX‐2 expression continued to increase in a time‐dependent manner from 4 to 24 h (Fig. 1C). A time course analysis of *COX‐2* mRNA revealed that its expression peaked between 8 and 12 h in RCM‐1 cells treated with DC alone, and returned to near baseline levels after 16 h (Fig. 1D, left).

Pretreatment with sts did not alter the time course of *COX‐2* mRNA expression; however, the peak level of *COX‐2* mRNA increased tenfold (Fig. 1D, right). The relative increases in the level of *COX‐2* mRNA in cells treated with DC or sts alone were not statistically different (14‐ and 15‐fold greater than with no treatment, respectively), whereas treatment with sts and DC resulted in a significant increase in the level of *COX‐2* mRNA, compared to that observed for the individual treatments (Fig. 1E). In HT‐29, a different cell line derived from colon carcinoma, the synergistic potentiation of DC‐induced COX‐2 expression by sts was also observed, indicating that this phenomenon is not specific to RCM‐1 cells (Fig. 1F).

**Figure 2.**
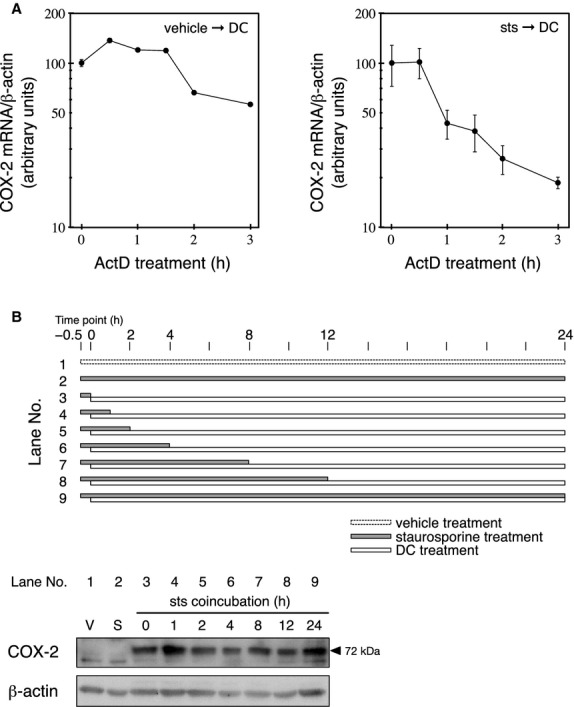
Effect of sts on *COX‐2 *mRNA stability and the irreversible potentiation of DC‐induced COX‐2 expression. (A) The RCM‐1 cells were pretreated with 10 nmol/L sts or vehicle control (0.05% DMSO) for 30 min, followed by treatment with 100 *μ*mol/L DC for 4 h. The cells were incubated in fresh medium containing 2 *μ*g/mL ActD for the indicated periods. *COX‐2 *mRNA levels were determined by real‐time PCR. Each data point represents the mean ± SE (bars) of a triplicate experiment. (B) RCM‐1 cells were pretreated with 10 nmol/L sts for 30 min, followed by a coincubation with 10 nmol/L sts and 100 *μ*mol/L DC for the indicated periods. Sts was removed by replacing the medium with fresh medium containing 100 *μ*mol/L DC and the incubation was continued until the total DC treatment time was 24 h. The RCM‐1 cells were also incubated with vehicle control (V) or 10 nmol/L sts only (S) for 24.5 h. The level of COX‐2 protein was determined by western blotting. The membrane was stripped, and then reprobed using an anti‐*β*‐actin antibody. The image is representative of three independent and reproducible experiments.

Previous studies have shown that DC enhances COX‐2 expression through both transcriptional and posttranscriptional mechanisms (Faour et al. 2001; Cao et al. 2007; Fernau et al. 2010). To examine whether the synergistic potentiation of COX‐2 expression was due to sts‐mediated mRNA stabilization, the degradation of *COX‐2* mRNA was assessed. Pretreatment with sts did not increase the stability of *COX‐2* mRNA, and an increase in degradation was observed between 1 h and 3 h after the initiation of treatment with the RNA synthesis inhibitor ActD (Fig. 2A).

We also examined the effect of varying the sts‐DC coincubation period on the increase in DC‐induced COX‐2 expression (Fig. 2B). The cells were pretreated in medium containing 10 nmol/L sts for 30 min before they were cultured in fresh medium containing both 10 nmol/L sts and 100 *μ*mol/L DC. At various intervals, the medium was replaced with fresh medium containing 100 *μ*mol/L DC only, and the cells were cultured for a total of 24 h before the level of COX‐2 protein was measured. The COX‐2 expression level observed after replacing the medium with DC immediately following the sts pretreatment (i.e., 0 h of coincubation) was higher than that observed in the absence of DC (sts treatment only; compare lanes 2 and 3 in Fig. 2B). Potentiated COX‐2 expression was also observed for the 1‐, 2‐, 4‐, 8‐, and 24‐h coincubation periods (Fig. 2B).

A synergistic increase in DC‐induced COX‐2 expression was also observed when sts‐pretreated cells (30 min) were incubated in a medium containing no sts or DC for 10 min before being treated with DC for 24 h (data not shown). These results suggest that the synergistic potentiation of COX‐2 expression occurs in two distinct stages. In the first stage, sts renders the RCM‐1 cells more sensitive to the effect of DC, and the cells exist in this state for a certain period after sts is removed from the culture medium. In the second stage, DC induces COX‐2 expression in the sensitized cells to a higher level than that induced by DC in the absence of sts.

**Figure 3.**
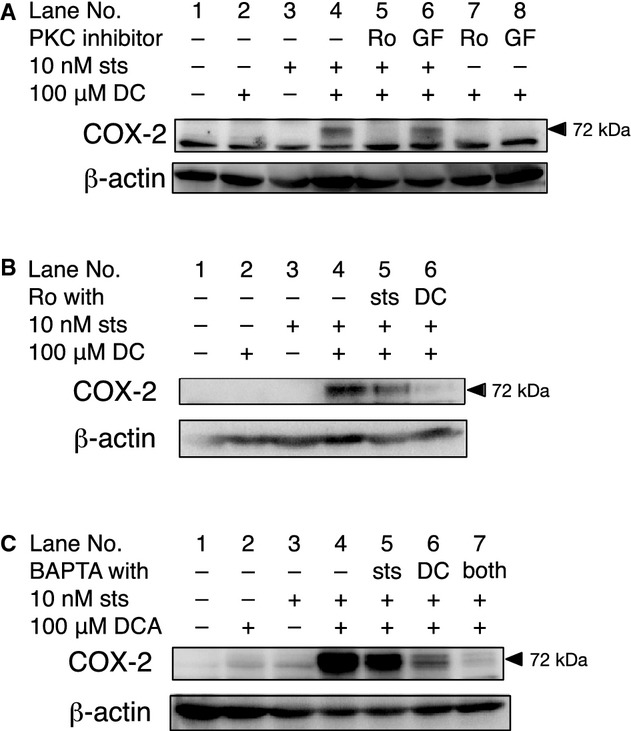
Roles of PKC and Ca^2+^ in the sts‐mediated synergistic potentiation of DC‐induced COX‐2expression. In each experiment, the posttreatment levels of the COX‐2 protein were determined by western blotting. The membranes were stripped, and then reprobed using an anti‐*β*‐actin antibody. The images are representative of at least three independent and reproducible experiments. (A) The RCM‐1 cells were pretreated with 5 *μ*mol/L Ro or 100 nmol/L GF in the presence or absence of 10 nmol/L sts for 30 min, followed by an incubation with 100 *μ*mol/L DC for 24 h. (B) The RCM‐1 cells were pretreated with 10 nmol/L sts with or without 5 *μ*mol/L Ro, followed by an incubation in fresh medium containing 100 *μ*mol/L DC with or without 5 *μ*mol/L Ro, as indicated. (C) The RCM‐1 cells were pretreated with 10 nmol/L sts with or without 30 *μ*mol/L BAPTA‐AM, followed by incubation in fresh medium containing 100 *μ*mol/L DC with or without 30 *μ*mol/L BAPTA‐AM for 24 h.

Sts has been shown to be a potent PKC inhibitor. Therefore, we investigated whether other PKC inhibitors synergistically potentiate DC‐induced COX‐2 expression. Pretreatment with the selective PKC inhibitors, Ro and GF produced no obvious effect on DC‐induced COX‐2 expression (Fig. 3A, lanes 7 and 8). However, we observed that pretreatment with Ro inhibited the sts‐mediated potentiation of DC‐induced COX‐2 expression (Fig. 3A lane 5). We further examined the stage at which Ro exerted this effect. COX‐2 expression was moderately reduced when RT was added simultaneously with sts in the 30‐min pretreatment period and then removed, while COX‐2 expression was more strongly inhibited when Ro was added simultaneously with DC following sts pretreatment (Fig. 3B). These results indicate that Ro inhibited the activity of DC in the second stage of sts‐mediated synergistic potentiation of DC‐induced COX‐2 expression.

We also investigated the role of Ca^2+^ in the sts‐mediated synergistic potentiation of DC‐induced COX‐2 expression. We observed that COX‐2 expression was inhibited in RCM‐1 cells incubated in a medium containing sts, DC, and the membrane‐permeable Ca^2+^ chelator, BAPTA‐AM. However, when the cells were pretreated with BAPTA‐AM and sts, the expression of COX‐2 was only moderately inhibited. These results suggest that Ca^2+^ is essential for DC‐induced COX‐2 expression, whereas sts does not require Ca^2+^ to sensitize cells to the effect of DC (Fig. 3C).

**Figure 4.**
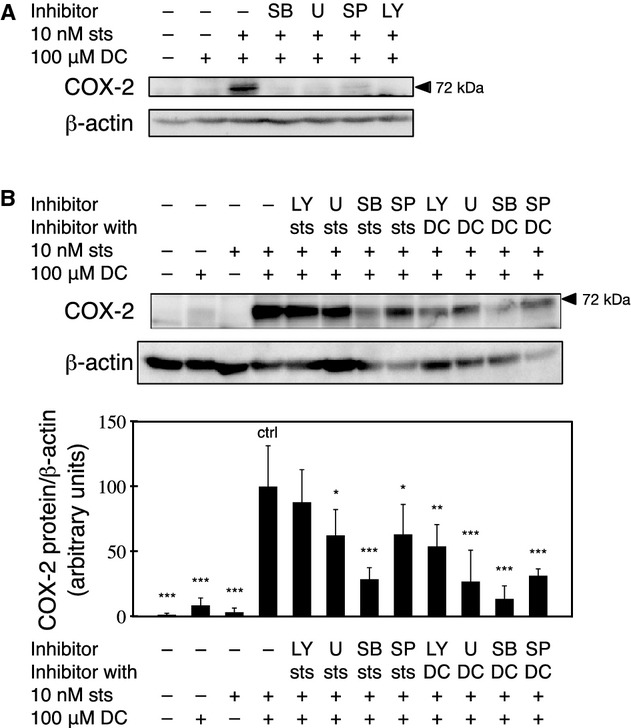
Analysis of signal transduction pathways involved in the sts‐mediated synergistic potentiation of DC‐induced COX‐2 expression. In each experiment, the posttreatment levels of the COX‐2 protein were determined by western blotting. The membranes were stripped, and then reprobed using an anti‐*β*‐actin antibody. (A) The RCM‐1 cells were pretreated with 10 nmol/L sts for 30 min in the presence or absence of 10 *μ*mol/L SB, 10 *μ*mol/L U0126 (U), 50 *μ*mol/L SP, or 20 *μ*mol/L LY, followed by an incubation with 100 *μ*mol/L DC for 24 h. (B) The RCM‐1 cells were pretreated with 10 nmol/L sts with or without the various kinase inhibitors for 30 min, followed by an incubation in fresh medium containing 100 *μ*mol/L DC with or without the various kinase inhibitors for 24 h, as indicated. The levels of COX‐2 protein were determined by western blotting. The relative intensities represent the mean ± SE of four independent experiments (*P < 0.05, **P < 0.01, and **^*^P < 0.0001, compared to no inhibitors [ctrl]).

To determine the signal transduction pathway involved in the sts‐mediated synergistic potentiation of DC‐induced COX‐2 expression, the effects of various kinase inhibitors were examined. The expression of COX‐2 in the RCM‐1 cells was inhibited when LY, U0126, SB, or SP was included in the medium containing sts and DC (Fig. 4A). However, when each inhibitor was added during the sts pretreatment only, the following effects on COX‐2 expression were observed: treatment with LY did not cause significant inhibition; treatment with U0126 or SP caused significant inhibition; and treatment with SB caused the greatest reduction in the level of the COX‐2 protein (Fig. 4B). For all of the inhibitors, a greater reduction in COX‐2 expression was observed when the inhibitor was added simultaneously with DC following sts pretreatment, compared to the effect of adding the inhibitor during sts pretreatment. However, the relative levels of inhibition among the four inhibitors were similar between their addition during and after pretreatment (Fig. 4B).

**Figure 5.**
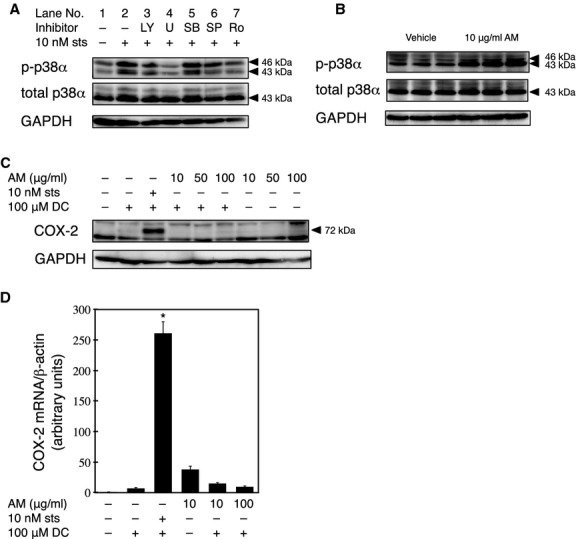
Role of p38 in the sts‐mediated synergistic potentiation of DC‐induced COX‐2 expression. (A) The RCM‐1 cells were treated with 10 nmol/L sts and 20 *μ*mol/L LY, 10 *μ*mol/L U0126, 10 *μ*mol/L SB, 50 *μ*mol/L SP, or 5 *μ*mol/L Ro for 30 min. The levels of phospho‐p38*α* (p‐p38*α*), total p38*α*, and GAPDH were sequentially determined by western blotting by stripping and reprobing a single membrane. (B) The RCM‐1 cells were treated with 10 *μ*g/mL anisomycin (AM) or vehicle control for 30 min. The levels of phospho‐p38*α* (p‐p38*α*), total p38*α*, and GAPDH were sequentially determined by western blotting by stripping and reprobing a single membrane. The experiment was performed in triplicate. (C) The RCM‐1 cells were pretreated with 10 nmol/L sts or 10, 50, or 100 *μ*g/mL AM for 30 min, followed by an incubation in fresh medium with or without 100 *μ*mol/L DC. The level of COX‐2 protein was determined by western blotting. The membrane was stripped, and then reprobed using an anti‐*β*‐actin antibody. (D) The RCM‐1 cells were pretreated with 10 or 100 *μ*g/mL AM or 10 nmol/L sts for 30 min, followed by an incubation in fresh medium with or without 100 *μ*mol/L DC. The levels of *COX‐2 *mRNA were determined by real‐time PCR, and are expressed as the mean ± SE (bars) of three independent experiments (*P < 0.05, compared to the vehicle‐treated cells).

Because SB caused the highest level of inhibition, we investigated the involvement of p38 in the sts‐mediated potentiation of DC‐induced COX‐2 expression. The level of phospho‐p38 in cells increased following a 30‐min treatment with sts (Fig. 5A, lane 2). To investigate whether the phosphorylation of p38 is sufficient for the sts‐mediated synergistic potentiation of DC‐induced COX‐2 expression, we performed a COX‐2 induction experiment using 10 *μ*g/mL (38 *μ*mol/L) anisomycin, an activator of stress‐induced MAPKs, in place of sts in the 30‐min pretreatment. Anisomycin treatment increased the level of phospho‐p38 in cells (Fig. 5B). However, pretreatment with 50 or 100 *μ*g/mL anisomycin did not increase the level of DC‐induced COX‐2 expression compared to that observed in cells treated with DC alone (Fig. 5C and D).

When each of the various signal transduction inhibitors was added simultaneously with sts, the level of phospho‐p38 was significantly lower in cells treated with U0126 than in cells treated with sts alone. The same effect was observed with Ro, albeit to a lesser extent (Fig. 5A lanes 3–7).

**Figure 6.**
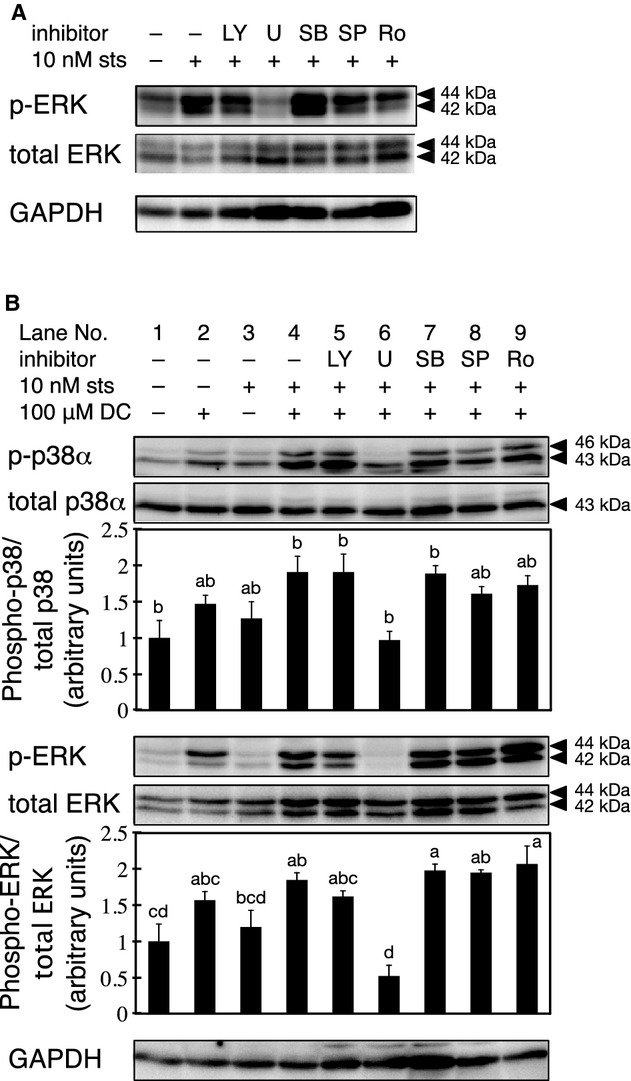
Effects of sts and DC on ERK phosphorylation. In each experiment, the expression levels of the various proteins posttreatment were sequentially determined by western blotting by stripping and reprobing a single membrane. (A) The RCM‐1 cells were treated with 10 nmol/L sts for 30 min in the presence of 20 *μ*mol/L LY, 10 *μ*mol/L U0126, 10 *μ*mol/L SB, 50 *μ*mol/L SP, or 5 *μ*mol/L Ro. The levels of phospho‐ERK (p‐ERK), total ERK, and GAPDH were determined by western blotting. (B) The RCM‐1 cells were pretreated with 10 nmol/L sts or vehicle control (0.05% DMSO) for 30 min, followed by incubation in fresh medium containing 100 *μ*mol/L DC with or without the various kinase inhibitors for 30 min. The levels of phospho‐p38 (p‐p38), total p38, phospho‐ERK (pERK), total ERK, and GAPDH were determined by western blotting. The relative intensities represent the mean ± SE of four independent experiments. Values not sharing a common character are significantly different (*P *< 0.05).

The reduction in p38 phosphorylation observed upon treatment with the MEK inhibitor U0126 prompted us to study the effects of sts on ERK activation. The level of phospho‐ERK was higher in cells treated with sts alone for 30 min than in cells treated with the vehicle control (Fig. 6A). However, adding U0126 to sts‐pretreated cells appeared to decrease the level of phospho‐ERK (Fig. 6A). We also examined the phosphorylation of ERK and p38 with and without sequential treatment with sts and DC for 30 min each. The level of phospho‐p38 tended to increase in cells treated with sts alone or DC alone, compared to that in the controls (Fig. 6B, p‐p38*α*, lanes 3 and 2, respectively).

Pretreatment with sts increased the phosphorylation of p38 in cells treated with DC (Fig. 6B, p‐p38*α*, lanes 2 and 4). However, the level of phospho‐p38 was significantly reduced when U0126 was added simultaneously with DC, whereas treatment with the other inhibitors produced no significant inhibitory effect (Fig. 6B, p‐p38*α*, lanes 5–9). In contrast, the level of phospho‐ERK was not increased in cells treated with sts alone for 30 min (Fig. 6B, p‐ERK, lane 3), and sts treatment had no detectable effect on the level of phospho‐ERK in DC‐treated cells (Fig. 6B, p‐ERK, lanes 2 and 4).

**Figure 7.**
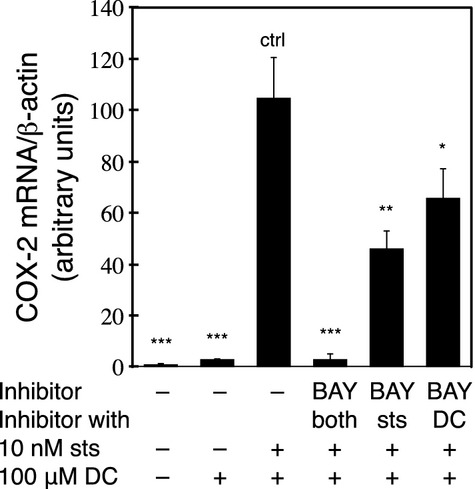
Role of NF‐*κ*B signaling in the sts‐mediated synergistic potentiation of DC‐induced COX‐2 expression. The RCM‐1 cells were pretreated with 10 nmol/L sts with or without 20 *μ*mol/L BAY for 30 min, followed by incubation in fresh medium containing 100 *μ*mol/L DC with or without 20 *μ*mol/L BAY for 24 h, as indicated. The levels of *COX‐2 *mRNA were determined by real‐time PCR, and are expressed as the mean ± SE (bars) of three experiments (*P < 0.05, **P < 0.0005, and **^*^P < 0.0001, compared to no BAY [ctrl]).

We also examined the effect of the NF‐*κ*B inhibitor, BAY, on sts in RCM‐1 cells to determine whether NF‐*κ*B signaling is involved in sts‐mediated synergistic potentiation of DC‐induced COX‐2 expression. The expression of COX‐2 was inhibited when the cells were treated with BAY along with sts and DC, indicating that the sts‐mediated synergistic potentiation of DC‐induced COX‐2 expression is dependent on NF‐*κ*B signaling. However, BAY inhibited COX‐2 expression more efficiently when it was added during the sts pretreatment than when it was added during the DC treatment alone (Fig. 7).

## Discussion

In our investigation of the signal transduction pathways involved in DC‐induced COX‐2 expression in the well‐differentiated rectum adenocarcinoma cell line RCM‐1, we discovered that pretreatment with sts potentiated DC‐induced COX‐2 expression. Because sts has been reported to inhibit bile‐acid‐induced COX‐2 expression (Zhang et al. 1998), the potentiating effect of sts was unexpected.

A previous study showed that 20–63 nmol/L sts induced COX‐2 expression in rat alveolar macrophages (Moon et al. 1995; Yamaki et al. 2000). In the current study, treatment of RCM‐1 cells with sts at concentrations ranging from 10 to 100 nmol/L did not produce statistically significant differences in *COX‐2* mRNA or protein expression levels compared to the vehicle control. However, the increased expression levels of *COX‐2* mRNA and protein resulting from cotreatment with sts and DC were higher than the sum of the expression levels observed after individual treatments with sts or DC. These results suggest that the effect of sts on DC‐induced COX‐2 expression is synergistic rather than additive.

The stabilization of *COX‐2* mRNA has been shown to contribute to tumorigenesis in the colon (Young et al. 2009). The effects of UVB, IL‐1*β*, and gastric acids on *COX‐2* mRNA stability and expression have also been reported (Faour et al. 2001; Souza et al. 2004; Fernau et al. 2010). However, in our experiments, pretreatment with sts did not increase the stability of *COX‐2* mRNA compared to that observed with DC treatment alone. It is conceivable that DC treatment increases the stability of *COX‐2* mRNA as a similarly hydrophobic bile acid, CDC, has been shown to increase the stability of *COX‐2* mRNA in intestinal epithelial cells (Zhang et al. 2000). However, it is likely that the increase in the level of *COX‐2* mRNA observed after cotreatment with sts and DC was due to the increased transcription rate of the *COX‐2* gene; thus sts probably had no additive effect on the stability of the *COX‐2* mRNA.

Sts is a potent inhibitor of PKC. We hypothesized that PKC signaling downregulates DC‐induced COX‐2 expression, and that sts blocks PKC‐mediated inhibition. To test this hypothesis, we examined the effect of the isoform‐selective PKC inhibitors Ro and GF on DC‐induced COX‐2 expression. However, no potentiation of DC‐induced COX‐2 expression was observed. On the contrary, when cells were pretreated with Ro and sts, DC‐induced COX‐2 expression was significantly reduced, whereas pretreatment with GF and sts had no significant inhibitory effect.

Previous studies have suggested that Ro is an effective inhibitor of PKC isozymes *α* and *ε*, whereas GF is an effective inhibitor of the *α*,* ε*, and *ζ* isozymes (Johnson et al. 1993). However, other studies have shown that Ro is a more effective inhibitor of PKC*ζ* than GF, with an IC_50_ value range of 103–169 nmol/L in pituitary cell extracts (Ison et al. 1993; Martiny‐Baron et al. 1993). Because we used 5 *μ*mol/L Ro in our experiment, it is likely that both nPKC and aPKC isoforms were inhibited by Ro to some degree. The in vitro IC_50_ values of GF for the PKC*α*/*β*/*γ*, PKC*δ*/*ε*, and PKC*ζ* isozymes have been reported as 10–20 nmol/L, 100–200 nmol/L, and 6 *μ*mol/L, respectively (Toullec et al. 1991; Martiny‐Baron et al. 1993), and other studies have reported an in vivo IC_50_ of 0.2–2 *μ*mol/L GF for the inhibition of PKC in cellular assays (Toullec et al. 1991; Heikkila et al. 1993). Because we used 100 nmol/L GF in our experiments, the contribution of PKC isozymes *α*,* β*,* δ*, or *ε* to sts‐mediated DC‐induced COX‐2 expression are likely excluded. Thus, our results suggest the involvement of PKC*η*, PKC*θ*, PKC*ι*, PKC*ζ*, or PKC*μ* (PKD).

The inhibitory effect of Ro on DC‐induced COX‐2 expression was moderate when it was added simultaneously with sts in the 30‐min pretreatment and then removed, whereas the effect was more intense when Ro was included in the DC treatment. Thus, although the DC‐mediated induction of COX‐2 expression is dependent on PKC activity, it is not essential for the effect of sts. Sts inhibits PKC through the competitive inhibition of ATP binding (Meggio et al. 1995), and stimulates the translocation of cPKC, nPKC (*δ* and *ε*), and aPKC (*ζ*) to the cell membrane (Wolf and Baggiolini 1988; O'Connell et al. 1997; Sawai et al. 1997). It is likely that the translocation of a subtype of PKC to the cell membrane is involved in the mechanism underlying the synergistic effect of sts on DC‐induced COX‐2 expression.

The results of our experiments using the membrane‐permeable Ca^2+^ chelator, BAPTA‐AM, suggest that Ca^2+^ is essential for the DC‐mediated induction of COX‐2 expression, but the effect of sts on DC‐induced COX‐2 expression is not dependent on Ca^2+^. Because the PKC isozymes implicated are Ca^2+^‐independent, a Ca^2+^‐dependent kinase is likely involved in DC‐induced COX‐2 expression.

In addition, sts has been reported to inhibit other protein kinases, such as cAMP‐dependent protein kinase, cGMP‐dependent protein kinase, Ca^2+^/calmodulin‐dependent protein kinase II, and myosin light chain kinase, at 1–20 nmol/L (Ruegg and Burgess 1989). Therefore, the possibility that sts exerts its potentiating effect on DC‐induced COX‐2 expression by inhibiting these kinases, which might downregulate COX‐2 expression, cannot be excluded.

Including the MAPK inhibitor SB in the sts pretreatment inhibited DC‐mediated COX‐2 expression, suggesting that p38 is critical to the action of sts. Thus, we examined whether phospho‐p38 is required to potentiate DC‐induced COX‐2 expression by treating RCM‐1 cells with anisomycin, an activator of stress‐induced MAPKs. Anisomycin has been shown to induce COX‐2 expression in the nontransformed intestinal epithelial cell line, IEC‐18, via the phosphorylation of p38 (Shafer and Slice 2005). In RCM‐1 cells, however, we observed that anisomycin treatment alone did not potentiate DC‐induced COX‐2 expression. Treatment with anisomycin alone caused a statistically insignificant increase in the level of *COX‐2* mRNA, and anisomycin pretreatment actually inhibited DC‐induced *COX‐2* mRNA expression. These results indicate that p38 phosphorylation is required for DC‐induced COX‐2 expression, but is not critical to the potentiating effect of sts.

The MEK inhibitor U0126 inhibited p38 phosphorylation, suggesting that the ERK pathway works upstream of the p38 pathway. However, when coincubated with either sts or DC, the inhibitory effect of U0126 on COX‐2 expression was less than that of SB. It seems contradictory that the effect of the upstream factor inhibitor was lower than that of the downstream factor inhibitor. However, given that sts stimulated ERK phosphorylation and U0126 intensely inhibited COX‐2 expression when included in both the sts and DC treatments, it is clear that the ERK pathway contributes to the sts‐mediated synergistic potentiation of DC‐induced COX‐2 expression.

The NF‐*κ*B pathway inhibitor BAY inhibited COX‐2 expression more efficiently when it was added during the sts pretreatment than when it was included in the DC treatment. Because the inhibitory effect of BAY on the cytokine‐induced phosphorylation of NF‐*κ*B inhibitor *α* is irreversible (Pierce et al. 1997), our results suggests that NF‐*κ*B is activated in the first stage or early in the second stage of sts‐mediated potentiation of DC‐induced COX‐2 expression. Rottlerin, a broad‐spectrum kinase inhibitor, a direct uncoupler of mitochondria, and a potent large conductance potassium channel opener (Davies et al. 2000; McGovern and Shoichet 2003; Zakharov et al. 2005; Balgi et al. 2009; Clements et al. 2011), has been shown to synergistically enhance the induction of COX‐2 expression by cytokines and inflammatory mediators (Park and Kwon 2011). However, a previous study showed that rottlerin‐ and IL‐1*β*‐induced COX‐2 expression were not inhibited by BAY or MG132 (Park and Kwon 2011), suggesting that the mechanism underlying the sts‐mediated synergistic potentiation of DC‐induced COX‐2 expression is in some way different from those of rottlerin and IL‐1*β*.

**Figure 8.**
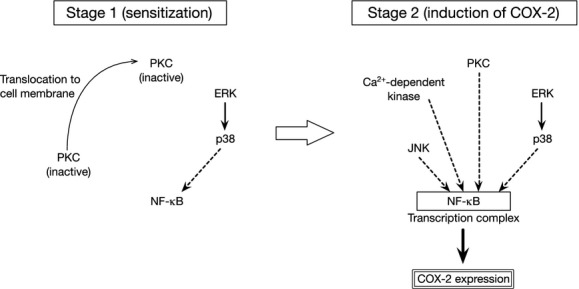
Schematic model of signaling pathways involved in the synergistic potentiation of COX‐2 expression. In the first stage (left), sts sensitizes the colon cancer cells to the effect of DC. Translocation of a PKC isozyme from the cytosol to the cell membrane is stimulated, and p38 is activated. The ERK pathway is predicted to act as an upstream factor of the p38 pathway. In the second stage (right), the DC‐mediated induction of COX‐2 expression is potentiated.

The results of our study indicate that sts synergistically potentiates DC‐induced COX‐2 expression through a process in which sts sensitizes cells to DC, thus enhancing the effects of DC on COX‐2 expression. Sts appears to act via the p38 pathway, but p38 phosphorylation alone is not sufficient to mimic the potentiating effect of sts on DC‐induced COX‐2 expression. The DC‐mediated induction of COX‐2 expression is apparently Ca^2+^ dependent. We believe that the sts‐mediated synergistic potentiation of DC‐induced COX‐2 expression involves signaling pathways mediated by PI3K, a canonical and stress‐inducible MAPK, a subtype of PKC (*η*,* θ*,* ι*,* ζ*, or *μ*), and NF‐*κ*B (Fig. 8). Our results suggest that bile‐acid‐mediated COX‐2 induction may be potentiated at higher levels than those previously reported if colorectal epithelial cells are sensitized by some kind of dietary or pharmacological substances. The elucidation of the underlying mechanism of sts‐mediated synergistic potentiation of COX‐2 induction is expected to contribute to the development of novel approaches for the prevention of tumorigenesis and tumor progression in colorectal cancer.

## Conflict of Interest

None declared.
